# DASFormer: self-supervised pretraining for earthquake monitoring

**DOI:** 10.1007/s44267-025-00085-y

**Published:** 2025-07-15

**Authors:** Qianggang Ding, Zhichao Shen, Weiqiang Zhu, Bang Liu

**Affiliations:** 1 Mila - Quebec AI Institute, University of Montreal, Montreal, Quebec H2S 3H1 Canada; 2 Woods Hole Oceanographic Institution, Woods Hole, MA 02543-1050 USA; 3 UC Berkeley Seismological Laboratory, Berkeley, CA 94720-4760 USA

**Keywords:** Earthquake monitoring, Image imputation, Time series forecasting, Self-supervised learning

## Abstract

Earthquake monitoring is a fundamental task to unravel the underlying physics of earthquakes and mitigate associated hazards for public safety. Distributed acoustic sensing, or DAS, which transforms pre-existing telecommunication cables into ultra-dense seismic networks, offers a cost-effective and scalable solution for next-generation earthquake monitoring. However, current approaches for earthquake monitoring like PhaseNet and PhaseNet-2 primarily rely on supervised learning, while manually labeled DAS data is quite limited and it is difficult to obtain more annotated datasets. In this paper, we present DASFormer, a novel self-supervised pretraining technique on DAS data with a coarse-to-fine framework that models spatial-temporal signal correlation. We treat earthquake monitoring as an anomaly detection task and demonstrate DASFormer can be directly utilized as a seismic phase detector. Experimental results demonstrate that DASFormer is effective in terms of several evaluation metrics and outperforms state-of-the-art time-series forecasting, anomaly detection, and foundation models on the unsupervised seismic detection task. We also demonstrate the potential of fine-tuning DASFormer to downstream tasks through case studies.

## Introduction

**Figure 1:**
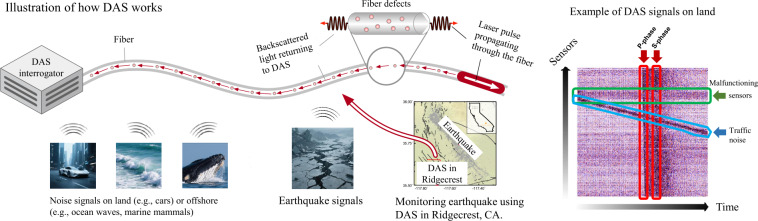
Illustration of how DAS works for earthquake monitoring. An example of DAS data collected in Ridgecrest City, CA is shown on the right

Earthquake, as a natural disaster, poses a constant threat to public safety due to its randomness and potentially catastrophic damage. Monitoring earthquakes using large networks of seismic sensors, such as seismometers, is a fundamental task to unravel the underlying physics and mitigate associated hazards [1]. Conventional seismic networks with sensor spacing of 10 to 100 km limit the capability of earthquake monitoring at finer scales. To overcome the bottleneck, a new technology called distributed acoustic sensing (DAS) offers a cost-effective and scalable solution [2–4]. DAS is an emerging technology that enables the monitoring of a wide range of physical phenomena, such as seismic events, pipeline leaks, and structural health such as the condition and integrity of buildings, bridges, tunnels, pipelines, and other critical infrastructure. Specifically, DAS systems use optical fibers as sensors to detect acoustic signals generated by the infrastructure, allowing for continuous and distributed monitoring of the structure’s condition. These sensors can be deployed over long distances, providing continuous and real-time monitoring of seismic activity over a large area. While DAS can detect early seismic waves and contribute to earthquake early warning systems, the lead time for warnings is often limited. However, it can still provide valuable seconds to minutes of advance notice, allowing for automated responses, such as slowing trains, shutting down industrial systems, or alerting emergency services. Figure 1 illustrates how DAS works: by sensing ground motion from back-scattered laser light due to fiber defects, DAS can transform ubiquitous telecommunication cables into ultra-dense monitoring networks. With thousands of seismic sensors in meter-scale spacing, DAS continuously records a wide range of natural signals, paving the way for next-generation earthquake monitoring [3, 4].

Recently, deep learning techniques have achieved progressive breakthroughs in extensive areas. Deep-learning-based methods [5–15] have been credited with significant advancements in earthquake monitoring. However, earthquake monitoring using DAS remains challenging due to the lack of manually labeled DAS data, especially P and S phase labels that carry vital information about seismic sources. Furthermore, it is a fundamental problem to generalize deep foundation models well for a wide range of downstream tasks, such as denoising and P/S phase picking. Current studies generally build on labeled or synthetic datasets [5, 7–15]. While some efforts have explored unsupervised learning, they remain limited in scope. For instance, j-DAS, an unsupervised method, has been developed, but it focuses exclusively on the denoising task [6], leaving other crucial tasks, such as phase picking, less explored.

Inspired by the success of BERT [16], a bidirectional transformer model for natural language processing that learns contextual representations through masked language modeling, we develop a novel self-supervised framework named DASFormer, which is pre-trained on a mask prediction task to enhance the representation ability for DAS data. For the ability for downstream tasks, we demonstrate that the well-pretrained DASFormer can be served as a time series detector for earthquake monitoring. The underlying assumption is that earthquakes occur randomly, making them significantly more difficult to predict causally over time compared to other sources, such as controlled explosions, industrial vibrations, or regular environmental noise, which often follow predictable patterns. Figure 1 shows an example that earthquake-generated P and S phases sharply appear as vertical anomalies on the DAS data whereas malfunctioning sensors and traffic signals have more temporal causality. Furthermore, we also demonstrate the potential of DASFormer on other downstream tasks like malfunctioning detection and semi-supervised P/S phase picking prediction. In our practical experiments, our method outperforms a variety of state-of-the-art time series forecasting, anomaly detection, and time series foundation models on realistic DAS data collected in Ridgecrest city, CA [17].

The contributions of this paper can be summarized as follows:

1. We propose a novel self-supervised framework for DAS data named DASFormer, which can be applied to downstream tasks for earthquake monitoring. DASFormer leverages the unique characteristics of DAS data to learn meaningful representations without the need for manually labeled data. By exploiting the temporal and spatial information captured by DAS, our framework can effectively capture the dynamic nature of seismic activities and extract valuable features for earthquake monitoring.
2. The model comes with a well-designed coarse-to-fine framework built on the top of Swin U-Net [18] and Convolutional U-Net [19] to effectively capture both spatial and temporal patterns in DAS data. To further enhance the framework, we introduce several key components such as preprocessing strategy, patching strategy, DASFormer blocks, and GAN-like training scheme [20].
3. We demonstrate the effectiveness of our method mainly on the task of unsupervised P/S phase detection on realistic DAS data. Our experimental results show that DASFormer outperforms 13 and 7 state-of-the-art methods for time series forecasting and anomaly detection, respectively, as well as 2 advanced time series foundation models. Furthermore, we also show the potential for other downstream tasks through case studies.

## Related work

### Deep learning on DAS data

Treating DAS data as time series with deep learning (DL) methods enhances sensitivity to small-magnitude earthquake signals compared to traditional techniques like STA/LTA [21] or template matching [22]. Notwithstanding, lacking precise arrival times for P/S phases remains a crucial challenge for the aforementioned methods. New deep-learning-based methods such as PhaseNet [7] and PhaseNet-DAS [23] used supervised U-Net networks for P/S phase picking when large datasets with manual labels are available; FMNet [9], EikoNet [10], and MarsQuakeNet [11] apply synthetic data to bypass label scarcity; *j*-DAS [6] offers a self-supervised framework only for signal denoising. This paper presents DASFormer, a self-supervised technique effective in both unlabeled and minimally labeled scenarios, with the potential for various downstream tasks. We present a summary of the differences between the mentioned methods using real DAS data in Table 1.

**Table 1:** Comparison of methods on DAS data. DASFormer stands out from other methods by offering a comprehensive suite of features

	Detect	Forecast	Without labels	Setting
PhaseNet	Yes	No	No	Supervised
PhaseNet-DAS	Yes	No	No	Semi-supervised
j-DAS	No	No	Yes	Unsupervised
DASFormer	Yes	Yes	Yes	Self-supervised

### Time series modeling

Temporal variation modeling, integral to time series analysis, has seen significant advancements recently. Classical methods such as the ARIMA families [24] were proposed based on prior assumptions of temporal variations. However, these fail to capture the non-stationarity and complexity of real-world time series. Consequently, deep learning-based models for time series have emerged, adapting MLPs [25–28] to time-series data and introducing temporal-aware neural networks such as TCN [29], RNN and LSTM [30]. Recently, Transformer [31] has garnered attention for time series modeling, with architectures like Informer [32], LogTrans [33], and ASTrans [34] addressing resource consumption issues. Autoformer [35] and FEDformer [36] focus on novel decomposition schemes for trend-cyclical component extraction in time series. TimesNet [37] proposes a task-general foundation model for time series analysis. Moreover, PatchTST [38] and ViTST [39] adapt the Vision Transformer (ViT) [40] for time series modeling, akin to our method. However, both of them use time series line graphs to illustrate temporal data points, while our method directly utilizes the magnitude of time points as values, leveraging the scalable window mechanism in Swin U-Net [18, 41, 42] to learn spatial and temporal patterns. Unlike previous methods that overlook the correlation and invariance in multi-variate time series like DAS data, our model considers spatial patterns in variates to complement temporal patterns, serving as a general foundation model.

### Anomaly detection

The P/S-phase detection task is a primary task in earthquake monitoring, which can be viewed as an anomaly detection problem. Anomaly detection methods typically fall into three categories: clustering-based, reconstruction-based, and forecasting-based methods. Clustering-based methods [43, 44] measure anomaly scores based on the sample’s distance to the cluster center. Reconstruction-based methods evaluate anomaly scores via the reconstruction error. Deep learning-based generative models like variational autoencoders (VAEs) [45–47] and GANs [48–51] have been widely investigated to generate reconstructed time series for anomaly detection. Recently, Anomaly Transformer [52] proposes to utilize the great sequential modeling capacity of Transformer [31] and renovate the self-attention mechanism specific to anomaly detection tasks. Forecasting-based methods [53, 54] typically leverage temporal models such as ARIMA and LSTMs to forecast time series, identifying anomalies based on the discrepancy between predicted and actual values. This paper illustrates how our base model can be used directly as a forecasting-based anomaly detection method for the P/S phase detection task in earthquake monitoring.

## Problem formulation of earthquake detection

In this paper, our main downstream task is the earthquake detection, also known as P/S phase detection, which is based on the DAS data, a kind of spatial-temporal multi-variate time series data. Formally, assume we have *N* DAS sensors that record measurements over a period of *T* time units. Let $\mathbf{X} \in \mathbb{R}^{N \times T}$ denote the DAS collected data matrix, with $\boldsymbol{X}_{i,j}$ being the measurement recorded by the *i*-th sensor at time *j*. The task of earthquake detection is to identify earthquake signals from background noise and other signals in the data matrix ***X***. The labels categorize each measurement $\boldsymbol{X}_{i,j}$ into binary $1 / 0$ indicating whether an earthquake signal is present.

**Figure 2:**
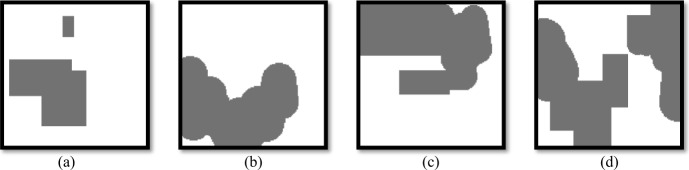
Samples of input masks used in DASFormer: (a) regular-only mask, (b) irregular-only mask, (c) and (d) a mix of regular and irregular masks. We use a mixed set of masks in practice

As a new technology, DAS has limited annotated earthquake labels. We tackle the lack of ground truth labels by considering an unsupervised setting in this paper. We train our model on a massive number of unlabeled raw DAS data, and evaluate it on a small labeled DAS signal subset. To effectively capture the temporal dynamics of DAS data, we pose this problem as a multi-variate time series forecasting task, which can be formulated as follows:

Given a multi-variate time series of DAS data from multiple sensors over a historical period *k* observed at the current time *t*, $\boldsymbol{I} = \{X_{:, t-k}, X_{:, t-k+1}, \ldots, X_{:, t}\}$, the goal is to train a model to predict the future time series in *p* time steps $\boldsymbol{O} = \{X_{:, t+1}, X_{:, t+2}, \ldots, X_{:, t+p}\}$. When an earthquake suddenly occurs, it generates seismic waves (i.e., P and S wave) that vibrate the fiber cables and are recorded by DAS. Figure 2 illustrates four examples of our regular and irregular masking patterns, which are combined to enhance the robustness of self-supervised representation learning. Regular masks follow a structured pattern, such as evenly spaced or block-based masking, while irregular masks introduce randomness or adaptive selection. By mixing both strategies, the model is exposed to diverse missing data scenarios, encouraging it to learn more generalizable and robust feature representations. This approach strengthens the model’s ability to capture meaningful patterns from incomplete inputs for downstream tasks.

## DASFormer

In this section, we introduce a self-supervised pretraining scheme on DAS data named DASFormer. Our approach is basically built on the top of Swin U-Net [18, 41] and Convolutional U-Net [19], and trained on the mask prediction task to learn the spatial-temporal representation of DAS data.

### DAS data preprocessing

DAS data can be typically represented as a time series with a large number of continuous variates (1250 in our datasets), which could be resource-consuming if we directly feed them into the model. To address this issue, we split the DAS data into segments based on its spatial-temporal invariance: (i) spatially uniform DAS sensor spacing and (ii) regular temporal sampling rate. Specifically, the raw DAS data are split into segments in size $V \times L$ with a stride of $\frac{V}{2}$ and $\frac{L}{2}$ alongside time and space dimensions, respectively, so that half of every two adjacent small blocks overlap.

### Architectural design of DASFormer

**Figure 3:**
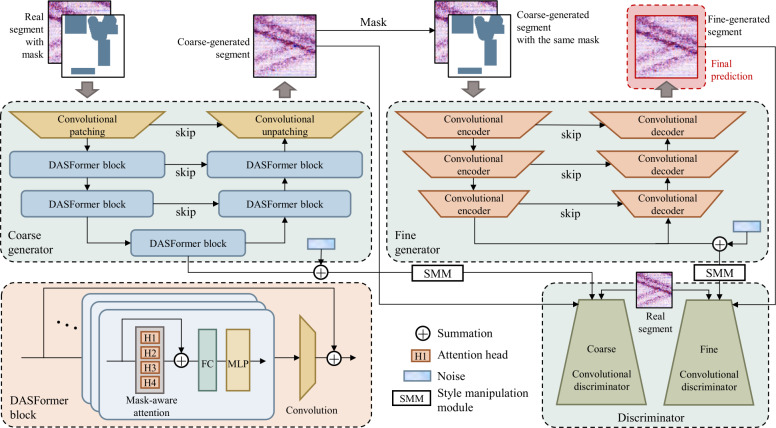
The pipeline of DASFormer with the coarse-to-fine framework including (i) Coarse Generator instantiated by Swin U-Net networks with DASFormer blocks, (ii) Fine Generator instantiated by Convolutional U-Net networks, and (iii) Noise-injected Discriminator instantiated by CNNs. For simplicity, here we omit patch-merging and patch-upsampling blocks between DASFormer blocks. Please refer to Fig. 4 for the detailed illustration of blocks

**Figure 4:**
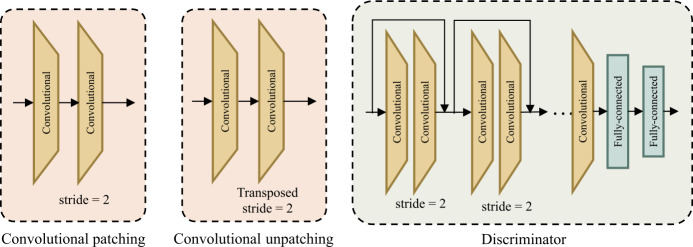
Detailed illustration of convolutional patching block (left), convolutional unpatching block (middle), and discriminator (right)

The overall pipeline of DASFormer is illustrated in Fig. 3, while a detailed view of its internal components is shown in Fig. 4.

#### Part 1: multi-variate time series masking

Inspired by pretrained language models like BERT [16], we mask a certain percentage of the input DAS data across both the temporal and variate dimensions in multi-variate time series. The goal is to learn robust and generalizable time series representations by predicting the masked data points from the context of the remaining data. Practically, we obscure values using a combination of masks with both regular and irregular shapes (see the input in Fig. 3).

Specifically, given a segment ***X*** in shape $V \times L$, our masking strategy entails selecting a data subset and applying a mask. This mask, a binary matrix ***M*** with the same shape $V \times L$ as the input segment ***X***, assigns the value of 1 to masked data points and 0 to unmasked points. Thus, our input comprises the segment ***X*** and the corresponding mask ***M***. We visualize four samples of our regular and irregular masks in Fig. 2, where regular masks follow a structured pattern, while irregular masks introduce random missing segments, simulating real-world data corruption. In practice, we use a mixed set of masks to improve robustness, ensuring the model learns to handle both structured and unstructured missing data effectively.

#### Part 2: overlapping convolutional patching

The non-overlapping linear patching strategy in the standard Swin Transformer is unsuitable for multi-variate time series data due to its neglect of temporal dependencies between adjacent patches, potentially disrupting local neighboring structures, particularly when discriminative regions are divided. To rectify this, we instead employ 2D convolutions to generate overlapping feature maps as patches, thereby capturing both temporal and spatial relationships. In this way, the input resolutions are not required to be strictly divisible by the predetermined patch size as the linear patching strategy. Formally, given the input in shape $V \times L$, we use two convolutional layers and produce $\frac{1}{2}$ sized feature maps as our patches.

#### Part 3: coarse-to-fine multi-variate time series generator

The DASFormer generator, designed to accurately predict missing data points, comprises two stages: a Swin U-Net-based network for coarse prediction and a U-Net network for fine prediction. This coarse-to-fine framework provides key advantages: (i) The coarse stage focuses on high-level features like contextual interaction in both temporal and spatial dimensions, while the fine stage refines predictions based on mask-specific detailed information, resulting in more realistic pretraining outcomes. (ii) The contextual knowledge acquired from the coarse stage can be transferred to downstream tasks, negating the need for training from scratch.

#### DASFormer block

To enable the model to dynamically update the mask state based on the unmasked data points, we propose the DASFormer block to adapt DAS time series data. Specifically, we revise the multi-head self-attention module in the standard Swin Transformer block so that attention is restricted to unmasked tokens only, making the model aware of available data while ignoring missing segments.

Additionally, the global nature of the vanilla attention mechanism lacks local temporal dependencies, which are crucial for DAS signals, as they exhibit highly localized patterns. To address this, we introduce an additional Gaussian mask $B_{h} $** to enhance the locality perception of the attention block, ensuring that attention scores favor nearby tokens.

Formally, the attention mechanism in our DASFormer block is formulated as follows:

1 $$\begin{aligned} &\boldsymbol{a}_{h} = \text{softmax} \left [ ( \frac{\boldsymbol{Q}_{h} \boldsymbol{K}_{h}^{\top}}{\sqrt{d_{k}}}) + \widetilde{\boldsymbol{M}} + \boldsymbol{B}_{h} \right ] \boldsymbol{V}_{h}, \end{aligned}$$

2 $$\begin{aligned} \text{where } &\widetilde{\boldsymbol{M}}_{i,j} = \textstyle\begin{cases} 0 &\text{if ${\boldsymbol{M}_{\text{token}}}_{i,j}=0$ } \\ -\text{inf} &\text{if ${\boldsymbol{M}_{\text{token}}}_{i,j}=1$ } \end{cases}\displaystyle , \end{aligned}$$

where $h = 1, \ldots, H $ denotes the *h*-th attention head, and $\boldsymbol{Q}_{h}$, $\boldsymbol{K}_{h}$, $\boldsymbol{V}_{h} $ are the Query, Key, and Value embeddings, respectively. The scaling factor $d_{k} $ represents the embedding dimension, while $\boldsymbol{M}_{\text{token}} $ denotes the masking matrix that dictates which tokens can attend to each other. The additional term $\boldsymbol{B}_{h} $ is a Gaussian locality constraint that biases the attention mechanism towards neighboring tokens.

The Gaussian bias $\boldsymbol{B}_{h} $ is computed as $(\boldsymbol{B}_{h})_{i, j} = - \frac{d\left (p(i), p(j)\right )}{2\sigma _{h}^{2}}$, where $p(i) = (i_{x}, i_{y}) $ is the 2D absolute position of token *i*, and $d(p(i), p(j)) = (i_{x} - j_{x})^{2} $ represents the 1D Euclidean distance between tokens along the time axis. By introducing this Gaussian bias, we enforce stronger attention scores to local regions. The masking term *M̃* ensures that the attention operation respects masked regions by preventing the model from attending to future or missing data. This is critical for self-supervised learning, where the model is trained to reconstruct missing segments only using available information. This mechanism is analogous to the causal mask in autoregressive models such as GPT, which enforces unidirectional attention to prevent information leakage from future or unvailable tokens.

**Figure 5:**
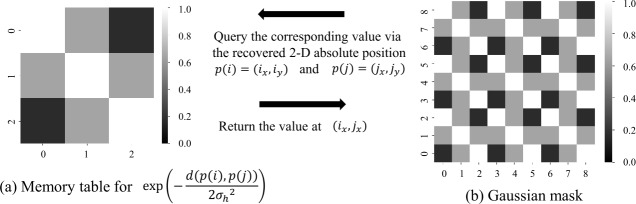
Illustration of Gaussian mask for better locality perception. For simplicity, we take a token space in size $3\times 3$ as an example, so the size of the Gaussian mask is $9 \times 9$ ($3^{2} \times 3^{2}$). The standard deviation $\sigma _{h}$ in the Gaussian distribution is set to $3/2$

We illustrate an example of Gaussian masking in Fig. 5, where the memory table stores the value of $\text{exp}\left (-\frac{d\left (p(i), p(j)\right )}{2\sigma _{h}^{2}} \right )$ at position $(p(i)_{x}, p(j)_{x})$, where $p(i) = (i_{x}, i_{y})$ the 2-D absolute position of token *i*, and $d(p(i), p(j)) = (i_{x} -j_{x})^{2}$ denotes 1-D Euclidean distance representing time distance between *i* and *j*. The value of Gaussian mask at position $(i,j)$ corresponds to the value of the memory table at position $(i_{x}, j_{x})$. Empirically, we apply Gaussian masking to 4 of 8 heads in our experiments, where the standard deviations $\sigma _{h}$ are set to $\{D/4, D/2, D, 2D\}$ for 4 heads, respectively, to learn multi-scale time dependencies, where *D* is the size of the time axis of the recovered 2-D token space.

#### Dynamic masking

Let $\boldsymbol{M}_{\text{token}}$ denote the token mask, and we define the initial token mask as $\boldsymbol{M}^{(0)}_{\text{token}} = \boldsymbol{M}$. In each training step, $\boldsymbol{M}_{\text{token}}$ is dynamically updated by an alternating strategy until all tokens are exposed as unmasked, meaning all masked tokens are learned and predicted. Specifically, in the *m*-th update, the mask $\boldsymbol{M}^{(m)}_{\text{token}}$ is obtained by applying a convolutional min-pooling over the recovered 2-D space of previous token mask $\boldsymbol{M}^{(m-1)}_{\text{token}}$ using a kernel in size $K \times K$ (with *K* being even) with stride *K*. In the next update, an extra $K/2$ padding size is applied to have overlapping update pairs, which makes the masked region gradually shrink and eventually disappear, i.e. eventually $\boldsymbol{M}_{\text{token}} = \mathbf{0}$.

**Figure 6:**
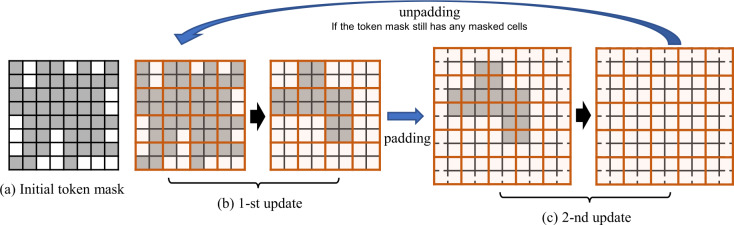
Illustration of the alternating updating strategy for dynamic masking in pre-training. The cells in grey and white mean masked and valid regions, respectively. For simplicity, the kernel size *K* is set to 2 in this case. As we can see, all tokens are finally exposed as valid after 2 updates

We illustrate an example of the alternating updating strategy for dynamic masking in Fig. 6, where the entire updating can be done in two steps. In each step, we apply a convolutional min-pooling operation over the mask. Usually, the number of updates can be much more than two; if so, we repeat the first and second updates shown in Fig. 6 in the following odd and even steps, respectively, until all tokens eventually become valid.

**Figure 7:**
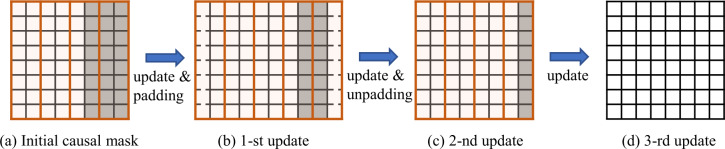
Illustration of the causal updating strategy for dynamic masking in forecasting. We shift a 1D window in size *K* alongside the temporal axis to causally update the token mask

For forecasting, the causal updating strategy for dynamic masking is illustrated in Fig. 7. By continually using a 1D convolutional min-pooling operation on the mask, the model is ultimately able to predict all the masked tokens.

#### Fine generator

We instantiate the fine generator with vanilla Convolutional U-Net networks [19], which takes the predicted segment from the coarse generator as input.

#### Part 4: noise-injected discriminator

DAS data includes various stochastic noise, such as environmental and instrumental noise. To mitigate this stochastic variation and enhance the diversity of generated data, we introduce stochastic noise into both (i) the feature activations after each convolutional layer and (ii) the convolutional weights themselves.

The discriminator in DASFormer is instantiated using stacked convolutional layers. Inspired by the StyleGAN family [55, 56], we employ a style manipulation module (SMM) to dynamically adjust the convolutional weights, reducing the model’s sensitivity to noise and promoting more diverse generations. The weight transformation process follows a two-step modulation:

3 $$\begin{aligned} \boldsymbol{s} &= f(\boldsymbol{C} + \boldsymbol{n}_{c}), \quad \boldsymbol{w}^{\prime }_{ijk} = \boldsymbol{s}_{i} \cdot \boldsymbol{w}_{ijk}, \end{aligned}$$

4 $$\begin{aligned} \boldsymbol{w}^{\prime \prime }_{ijk} &= \boldsymbol{w}^{\prime }_{ijk} \left / \sqrt{\sum _{i,k} { \boldsymbol{w}^{\prime }_{ijk}}^{2} + \epsilon} \right . , \end{aligned}$$

where $\boldsymbol{n}_{c} \sim \mathcal{N}(0, \sigma _{c}^{2}\mathbf{I}) $ represents a Gaussian noise term injected into the style code ***C*** learned from the generator, ***s*** is the modulated style vector which controls the adaptive transformation of convolutional weights. $\boldsymbol{w}_{ijk} $ denotes the original convolutional weight for the *i*-th filter, *j*-th input channel, and *k*-th output channel. $\boldsymbol{w}^{\prime }_{ijk} $ is the intermediate weight transformation, incorporating style-based modulation. $\boldsymbol{w}^{\prime \prime }_{ijk} $ is the final normalized weight, ensuring numerical stability and balanced gradient flow during training.

This two-step weight modulation mechanism allows the model to dynamically adjust feature representations, making it more resilient to noise while enhancing the diversity of generated outputs.

#### Objective function

Due to the presence of random noise in DAS data, it is not appropriate to evaluate the generation quality by measuring the point-wise error like MSE. Instead, we minimize the error of high-level feature maps rather than raw signals as the training objective of the generator. Specifically, we train an extra VGG-based autoencoder [57] on DAS data using a reconstruction loss and utilize its encoder as a high-level feature extractor. The overall objective function for both the coarse and fine generators is formulated as:

5 $$\begin{aligned} \operatorname*{argmin}_{\theta _{g}} \; & \mathcal{L}(\theta _{g}, \boldsymbol{X}) + \alpha \left \|\nabla _{\boldsymbol{X}} \mathcal{L}( \theta _{g}, \boldsymbol{X})\right \| + \beta \mathcal{L}_{G} + \left \|\theta _{g}\right \|, \end{aligned}$$

6 $$\begin{aligned} \text{where } & \mathcal{L}(\theta _{g}, \boldsymbol{X})= \sum _{i} \left \|\phi _{i} (\hat{\boldsymbol{X}}) - \phi _{i}(\boldsymbol{X}) \right \|_{1}, \end{aligned}$$

where $\phi _{i}(\cdot ) $ denotes the *i*-th feature map extracted from the pretrained encoder, $\theta _{g} $ represents the trainable parameters of the generator. $\hat{\boldsymbol{X}} $ is the generated segment. $||\nabla _{\boldsymbol{X}} \mathcal{L}(\theta _{g}, \boldsymbol{X})|| $ is a gradient regularization term ensuring that the model’s gradients do not grow excessively, thereby stabilizing training [58]. $\mathcal{L}_{G} $ is the adversarial loss from the GAN framework [20], encouraging realistic reconstruction. $||\theta _{g}|| $ represents a weight regularization term to mitigate overfitting. Here $||\cdot ||_{1} $ denotes the L1 norm, which measures the absolute difference between predicted and real feature maps. $||\cdot || $ generally represents the L2 norm when applied to weight regularization, ensuring controlled parameter updates. The agorithm view of DASFormer is shown in Alg. 1.

**Algorithm 1:**
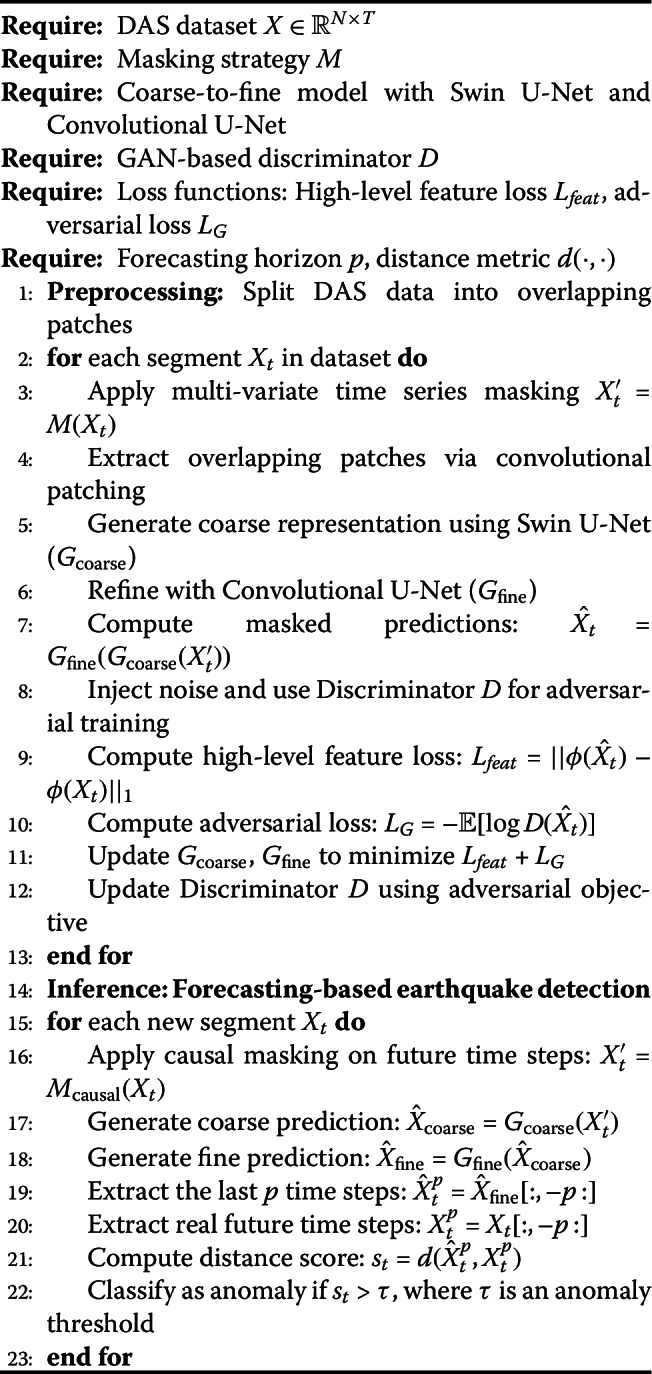
DASFormer: Self-supervised pretraining and forecasting-based earthquake detection

### Apply DASFormer to downstream tasks

#### Earthquake detection

DAS signals comprise ubiquitous, uniform random noise and environmental noises like traffic, which exhibit explicit temporal patterns due to repetitive or cyclical processes. Conversely, P and S signals are influenced by unpredictable events like earthquakes, resulting in complex and irregular patterns. We can directly utilize DASFormer as a forecasting-based anomaly detector, capable of identifying irregular patterns that significantly deviate from predicted values, especially in the absence of ground-truth earthquake event labels.

**Figure 8:**
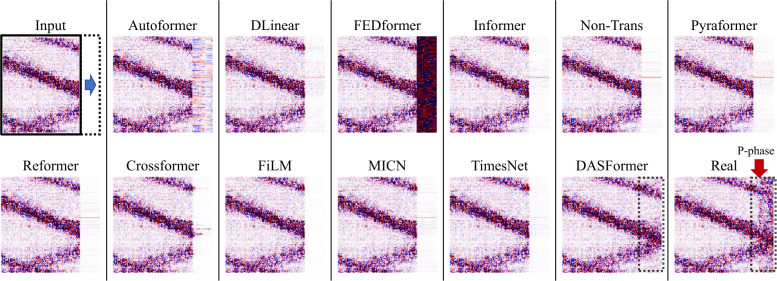
Visual comparison between forecasting methods. The dashed frame and the blue arrow denote the forecasting window and direction, respectively. The red arrow in the Real panel indicates an earthquake signal (P-phase) in real data. Note that our DASFormer successfully predicts the temporal trends of background signals while all baselines fail. Therefore, the earthquake can be easily exposed by measuring the distance between the forecasting region of Real and DASFormer panels

To derive predicted values, we introduce a causal mask (only mask the tail *p* time steps of the segment, see inputs in Fig. 8). To have causal forecasting, we shift a 1-D window in size *K* alongside only the time axis instead of the 2-D window in dynamic masking.

#### Other downstream tasks

As a pre-trained model, DASFormer can be naturally fine-tuned to adapt to arbitrary downstream tasks. Typically, we take the coarse part of DASFormer, the Swin U-Net-based blocks, as the feature extractor, and fine-tune the subsequent convolutions. Take the task of precise (point-level prediction) P/S phase picking as an example, we first freeze the blocks, then change the dimension of the output layers and apply the point-wise classification loss function, and finally re-train the DASFormer in a supervised-learning setting.

## Experiments

### Experimental setup

#### Datasets

We conduct experiments using DAS data collected by Caltech in Ridgecrest city, California [17] as shown in Fig. 1, of which one month of data is publicly available via Southern California Earthquake Data Center [59].^1^ The Ridgecrest DAS transforms a 10 km telecommunication cable into 1250 sensors in 8 m spacing and records the continuous data at 250 Hz sampling rate. We follow the filtering and cleaning procedure introduced in PhaseNet [7] to preprocess the raw signals. We split 90 earthquake events into training/validation/testing sets alongside the timeline with 45/20/25 events, respectively. Then, we downsample the cleaned signals to 10 Hz and cut the DAS signals into $128\times 128$ segments with a stride of 64 steps using the preprocessing scheme aforementioned in Sect. 4.1. After that, we obtain 31,464 segments in total and 21,672/4352/5440 segments in training/validation/testing sets pertaining on the mask prediction task. The segmentation for evaluating the performance on the P/S phase detection task is different and will be introduced later.

We only use segments in the testing set to evaluate the performance of DASFormer and all baselines. Specifically, we adopt a shift window in $128\times 128$ with a stride of 10 time steps (1 second) to obtain the segments. After that, we have $14,688$ segments in total for evaluation.

We show the statistics of datasets in Table 2. In addition to the datasets on land, we also incorporate a dataset in submarine. Between Nov. 1st and 5th, 2021, two fiber-optic backbone cables were temporarily converted to submarine DAS arrays (referred to as OOI North and OOI South) [60]. In this study, we use the OOI North as an example. It was connected to an Optasense QuantX interrogator to continuously record ground vibrations up to the first optical repeater located at ∼65 km from the shore. With a sensor spacing of ∼2 m, the OOI North array has a total of 32,600 sensors.^2^ The preprocessing strategy of submarine datasets is the same as before.

**Table 2:** Statistics of datasets

Descriptions	Training	Validation	Testing	Evaluation
# of seconds	11,370	1800	2250	2250
# of sensors	1250	1250	1250	1250
Frequency rate	10 Hz	10 Hz	10 Hz	10 Hz
# of earthquakes	45	20	25	25
# of segments	21,672	4352	5440	14,688
Size of segments	128 × 128	128 × 128	128 × 128	128 × 128
Stride of segments	64	64	64	10

#### Benchmarks

To demonstrate the superiority of the proposed DASFormer method, we extensively select 13 time series forecasting methods, 7 anomaly detection methods, and 2 foundation model-based methods as the benchmarks in this paper, including LSTM [61], TCN [62], Transformer [31], Crossformer [63], Reformer [64], Pyraformer [65], Nonstationary Transformer (Non-Trans) [66], Informer [32], FEDformer [36], DLinear [27], Autoformer [35], MICN [67], and FiLM [68] for forecasting-based methods, and LSTM-VAE [69], LSTM-GAN [48], ConvNet-VAE [70], ConvNet-GAN [48], Deep-SVDD [44], GDN [71], and Anomaly Transformer (Anomaly-Trans) [52] for anomaly detection-based methods. For forecasting-based methods, we use the distance between the predicted values and the real values in the forecasting window as the anomaly score. For anomaly detection-based methods, the anomaly score is the distance between the reconstructed values and the observed values in the forecasting window. For Deep-SVDD, GDN, and Anomaly Transformer, we further show the results with their specific distances as anomaly scores. We also involve two foundation models, TimesNet [37] and *j*-DAS [6]. Two traditional methods originated in PhaseNet [7] are included as baselines.

#### Implementation details & metrics

For the earthquake detection task, we adopt a shift window with a smaller stride of 10 time steps (1 second) and predict the next 10 time steps to have non-overlapped forecasting alongside the time axis. We use window-wise labels instead of point-wise labels for a more robust evaluation. A forecasting region is designated as an anomaly if it contains at least one annotated anomaly data point. An anomaly score is defined by the distance between the realistic signal values and the predicted signal values within the forecasting region. We investigate several distance functions in practice, including absolute error with Euclidean distance (AE), Earth Mover’s Distance (EMD) [72], and sliced EMD [73]. We calculate metrics, such as ROC-AUC and F1-score, for this binary classification.

For P/S phase picking task, we adopt convolutions on the top of the DASFormer blocks of the coarse generator and then fine-tune these convolutions with a point-wise loss function on the labeled DAS data. To make the model more robust to the signals, following PhaseNet [7], we soften the point-wise hard-encoded labels (1 for P/S phases, 0 for others) to values ranging from 0 to 1 by a Gaussian distribution.

### Results on Ridgecrest datasets

We compare our method with the aforementioned baselines on the earthquake detection task, and the results are presented in Table 3. Our DASFormer achieves the highest performance across all evaluation metrics, significantly outperforming all baselines in both ROC-AUC score and F1 score. Additionally, we observe that both AE and sliced EMD serve as appropriate distance functions for this task. To ensure a fair comparison, we report the best and second-best results consistently for AUC and F1-score across all methods, rather than ranking performance under different distance functions independently.

**Table 3:** Complete comparison results between state-of-the-art methods of time series forecasting, anomaly detection, and foundation models. We measure the distance by absolute error (AE), Earth-Mover Distance (EMD), and sliced EMD. For forecasting methods, the look-back and forecasting window sizes are set to 100 and 10, respectively. Method-specific denotes the distance proposed by the specific method. For reconstruction methods, we reconstruct all 110 time steps and use the last 10 for distance measuring. We use ROC-AUC score (AUC) and F1 score (F1) as metrics. The best and second-best results are in bold and underlined, respectively. ‘-’ denotes not available

Methods	Distance	AE		EMD		Sliced EMD		Method-specific	
	Metric	AUC	F1	AUC	F1	AUC	F1	AUC	F1
Traditional method	Aggregation-0	0.730	0.246	0.692	0.277	0.709	0.246	-	-
	Aggregation-inf	0.509	0.014	0.501	0.006	0.497	0.009	-	-
Forecasting	LSTM	0.726	0.243	0.691	0.277	0.709	0.253	-	-
	TCN	0.730	0.240	0.701	0.277	0.7	0.253	-	-
	Transformer	0.736	0.245	0.695	0.276	0.702	0.251	-	-
	Crossformer	0.730	0.246	0.702	0.275	0.712	0.251	-	-
	Reformer	0.730	0.247	0.694	0.277	0.709	0.253	-	-
	Pyraformer	0.726	0.245	0.693	0.278	0.707	0.254	-	-
	Non-Trans	0.727	0.238	0.697	0.277	0.710	0.252	-	-
	Informer	0.729	0.241	0.698	0.275	0.709	0.252	-	-
	FEDformer	0.522	0.071	0.492	0.130	0.508	0.131	-	-
	DLinear	0.729	0.233	0.690	0.272	0.703	0.247	-	-
	Autoformer	0.727	0.233	0.691	0.265	0.707	0.246	-	-
	MICN	0.739	0.247	0.699	0.277	0.705	0.256	-	-
	FiLM	0.735	0.245	0.694	0.278	0.702	0.250	-	-
Anomaly detection	LSTM-VAE	0.727	0.241	0.698	0.273	0.707	0.251	-	-
	LSTM-GAN	0.732	0.245	0.697	0.277	0.705	0.252	-	-
	ConvNet-VAE	0.734	0.247	0.701	0.280	0.711	0.257	-	-
	ConvNet-GAN	0.724	0.244	0.695	0.270	0.707	0.249	-	-
	Deep-SVDD	0.726	0.234	0.694	0.277	0.702	0.251	0.564	0.176
	GDN	0.732	0.247	0.698	0.279	0.708	0.255	0.618	0.114
	Anomaly-Trans	0.730	0.245	0.694	0.275	0.710	0.252	0.543	0.156
Foundation model	TimesNet	0.733	0.243	0.699	0.277	0.708	0.255	-	-
	*j*-DAS	0.754	0.249	0.703	0.272	0.698	0.263	-	-
	Dasformer (ours)	0.886	0.552	0.890	0.527	**0.906**	**0.565**	-	-

Table 3 demonstrates that DASFormer outperforms all state-of-the-art forecasting, anomaly detection, and foundation models. Specifically, DASFormer achieves the highest AUC of 0.906 and F1 of 0.565 under sliced EMD, confirming that DASFormer excels in detecting fine-grained seismic activities.

#### Failure of forecasting-based methods

The visual comparison in Fig. 8 reveals that all forecasting-based baselines fail to predict DAS signals accurately. This can be attributed to the following limitations:

(i) Noisy signals. DAS data contains significant environmental and instrumental noise, leading to numerous spurious signals that disrupt training. Forecasting-based models rely heavily on point-wise loss functions, which are highly sensitive to noise, making them ineffective for seismic anomaly detection.

(ii) Lack of spatial awareness. Most baselines treat different variates as independent time series, failing to capture the spatial coherence of DAS data. Some methods attempt to integrate graph neural networks (GNNs) to model spatial correlations; however, they fail to accurately represent the propagation characteristics of seismic waves, which follow a structured spatial-temporal pattern.

#### Failure of reconstruction-based methods

These models also fail to achieve competitive results due to the following reasons:

(i) Lack of causal awareness. In earthquake monitoring, P-phase and S-phase signals are inherently unpredictable. However, reconstruction-based models do not enforce causal constraints, leading to unrealistic reconstructions that do not align with real seismic events.

(ii) Limited scalability for large anomalies. Generative models such as ConvAE, VAE, and GAN are designed to detect small-scale anomalies but fail to capture large, long-duration P-phase and S-phase events, resulting in suboptimal anomaly detection.

#### Superior performance of DASFormer

**Figure 9:**
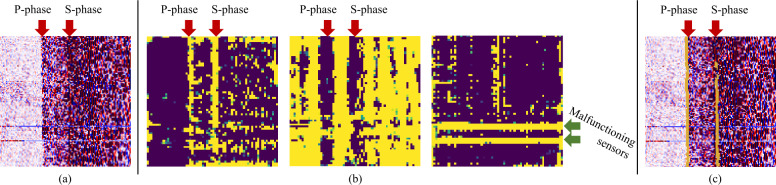
Extracted features of DASFormer: (a) The input DAS signals. (b) Three selected feature maps extracted from the last DASFormer block of the pretrained DASFormer before fine-tuning. (c) The P/S phase picking results of DASFormer in a semi-supervised setting trained with only 20 labeled samples

DASFormer overcomes these challenges due to:

(i) High-level feature reconstruction. Instead of relying on raw signal differences, DASFormer optimizes reconstruction at the feature level, ensuring robustness to noise. We illustrate the extracted features in Fig. 9 for more details.

(ii) Swin U-Net and Convolutional U-Net for spatial-temporal learning. The combination of transformer-based hierarchical feature extraction and localized convolutional refinement enables DASFormer to capture seismic wave propagation more effectively.

(iii) Causal forecasting-based anomaly detection. Unlike traditional reconstruction-based models, DASFormer enforces causal constraints, ensuring that anomaly detection remains temporally consistent with real seismic events.

These design choices allow DASFormer to significantly outperform both forecasting-based and reconstruction-based baselines, demonstrating its effectiveness in earthquake monitoring.

### Results on submarine datasets

Except for the Ridgecrest DAS example on land, DAS is also promising for deploying large-aperture and long-term monitoring networks at logistically challenging places. For instance, deploying DAS in the harsh ocean. We take advantage of a four-day community submarine DAS experiment offshore central Oregon [60] to examine the robustness of our DASFormer.

**Figure 10:**
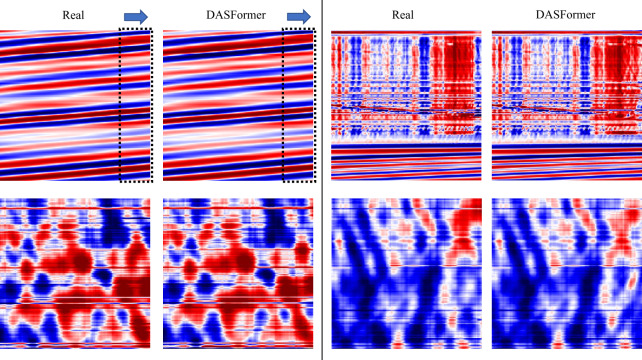
Visualization of applying DASFormer to submarine DAS data. The dashed frame and the blue arrow in the first case denote the forecasting window and direction, respectively, which are omitted in the rest of cases for clarity

We pre-train an Ocean-DASFormer on this submarine DAS data and visualize four cases in Fig. 10:

1. (Upper left) Ocean gravity waves.
2. (Upper right) Land-ocean boundary (land on top and ocean at bottom).
3. (Lower left) and (Lower right) Noise in the ocean at different water depths.

Ocean-DASFormer effectively captures the spatial and temporal patterns of all examples in various environments, indicating its potential for submarine DAS data in the future.

## Analysis

### Results with confidence intervals

Table 4 presents the average performances and $\pm 95\%$ confidence intervals with five individual runs. As we can see, the small gaps in the confidence intervals across the five individual runs illustrate the robustness and consistency of our method.

**Table 4:** Results with $\pm 95\%$ confidence intervals ($1.959 \times \sigma $)

Distance	Metrics	
	AUC	F1
AE	0.886 ± 0.0028	0.552 ± 0.0008
EMD	0.890 ± 0.0025	0.527 ± 0.0008
Sliced EMD	0.906 ± 0.0039	0.565 ± 0.0006

Despite the inherent randomness in DAS data and potential variations in each run, our method managed to maintain a relatively stable performance, as indicated by the narrow confidence intervals. This level of stability serves as a strong indication that our model is not overly sensitive to the specificities of the randomness in the training process.

### S/P phase analysis

The results for P-phase and S-phase detection are shown in Table 5. Our method performs better on S-phase than P-phase, which is within our expectations as earthquakes typically generate stronger S-phase due to their underlying physics. Intriguingly, AE and sliced EMD demonstrate superior performance on P-phase and S-phase, respectively. So in practice, we recommend the use of different distance functions for detecting P-phase and S-phase.

**Table 5:** The results for P and S phases separately. The best results for each phase are in bold

Phase	AE		EMD		Sliced EMD	
	AUC	F1	AUC	F1	AUC	F1
P-phase	**0.860**	**0.335**	0.830	0.302	0.843	0.327
S-phase	0.908	0.577	0.947	0.561	**0.959**	**0.586**
Both	0.886	0.552	0.890	0.527	**0.906**	**0.565**

### Ablation study

#### Role of proposed modules

To explore the role of each module in our proposed framework, we conduct ablation studies with the following comparisons: (i) convolutional patching v.s. linear patching, (ii) DASFormer block v.s. vanilla Swin Transformer blocks, and (iii) high-level encoder-based generative loss v.s. point-wise MSE generative loss. The results are shown in Table 6, where we conclude that all of the proposed modules could strengthen the performance of the model consistently. We also conduct ablation studies on the quantity of training data and architectural components to investigate the amount of training data required for efficient seismic monitoring and the individual contributions of each component of our architecture, respectively.

**Table 6:** Comparison results for ablation studies

	AE		EMD		Sliced EMD	
	AUC	F1	AUC	F1	AUC	F1
Replace convolutional patching w/ linear patching	0.826	0.435	0.770	0.341	0.663	0.299
Replace DASFormer w/ Swin-Trans block	0.869	0.428	0.782	0.335	0.748	0.316
Replace high-level loss w/ Point-wise MSE loss	0.880	0.435	0.839	0.499	0.743	0.314
Remove noise injection in Discriminator	0.856	0.523	0.857	**0.540**	0.830	0.443
DASFormer (Ours)	**0.886**	**0.552**	**0.890**	0.527	**0.906**	**0.565**

#### Amount of training data

We also conduct ablation experiments to have a better understanding of the amount of training data required for efficient seismic monitoring. In detail, we vary the size of the training dataset to examine its impact on the performance of our method. Our full DAS training data contains 45 earthquake events. We reduce the number of earthquake events from 45 to 5, in stages of 45, 20, 10, and 5, and examine the corresponding performance. The results are shown in Table 7. It is worth noting that the model maintains similar performance levels with just 20 events. However, a significant drop in performance is noted when the event number is reduced to 10. The findings from the experiments indicate that our pretrained model does not necessitate an extensive amount of data to yield satisfactory results.

**Table 7:** Ablation study on quantity of training data

	AE		EMD		Sliced EMD	
	AUC	F1	AUC	F1	AUC	F1
5 events	0.71	0.34	0.74	0.37	0.72	0.37
10 events	0.78	0.37	0.80	0.44	0.80	0.42
20 events	0.86	0.52	0.88	0.51	0.88	0.54
45 events (Full data)	0.88	0.55	0.89	0.52	0.90	0.56

#### Architectural components

Remember that our model is composed of a coarse generator, a fine generator, and a discriminator. To examine the individual contributions and effectiveness of each component, we perform an ablation study on the three stages (Coarse, Coarse-to-Fine, Coarse-to-Fine with Discriminator). As shown in Table 8, the integration of the fine generator and the discriminator enhances the results, thereby validating the efficacy of the coarse-to-fine framework and the discriminator.

**Table 8:** Ablation study on architectural components

	AE		EMD		Sliced EMD	
	AUC	F1	AUC	F1	AUC	F1
Coarse-only	0.79	0.41	0.81	0.44	0.83	0.47
Coarse-to-Fine	0.85	0.52	0.87	0.53	0.87	0.50
Coarse-to-Fine w/ GAN	0.88	0.55	0.89	0.52	0.90	0.56

### Signal-to-noise (SNR) analysis

**Figure 11:**
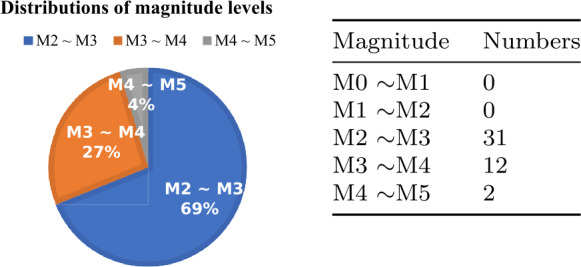
The distribution of magnitude levels in the training set of our DAS datasets

In this section, we discuss our findings with the magnitude levels of earthquakes. We show the magnitude distribution of the training set of our datasets in Fig. 11. Generally speaking, earthquakes with higher magnitudes have higher amplitude seismic waves, which means a higher signal-to-noise ratio (SNR) because of the stronger signals (seismic waves).

Table 9 illustrates our findings, comparing our method with Aggregation-0 across different earthquake magnitude levels. Our method shows improved performance as the magnitude of earthquakes increases, particularly in the M2 ∼ M3 and M3 ∼ M4 ranges. In each of these categories, DASFormer outperformed Aggregation-0. It is worth noting that for earthquakes within the M0 ∼ M2 and M4 ∼ M5 ranges, the sample size is insufficient to evaluate.

**Table 9:** The distribution of magnitude levels in the testing set of our DAS datasets and the experimental results across different magnitudes. For each pair, DASFormer is on the left side and Aggregation-0 (baseline) is on the right side

Magnitude	Numbers	AE		EMD		Sliced EMD	
		AUC	F1	AUC	F1	AUC	F1
M0 ∼M1	0	N/A	N/A	N/A	N/A	N/A	N/A
M1 ∼M2	1	N/A	N/A	N/A	N/A	N/A	N/A
M2 ∼M3	27	0.84/0.74	0.40/0.24	0.88/0.70	0.46/0.28	0.86/0.70	0.42/0.25
M3 ∼M4	14	0.89/0.73	0.59/0.24	0.92/0.69	0.58/0.26	0.93/0.70	0.60/0.25
M4 ∼M5	3	N/A	N/A	N/A	N/A	N/A	N/A

## Case studies

### Additional cases

**Figure 12:**
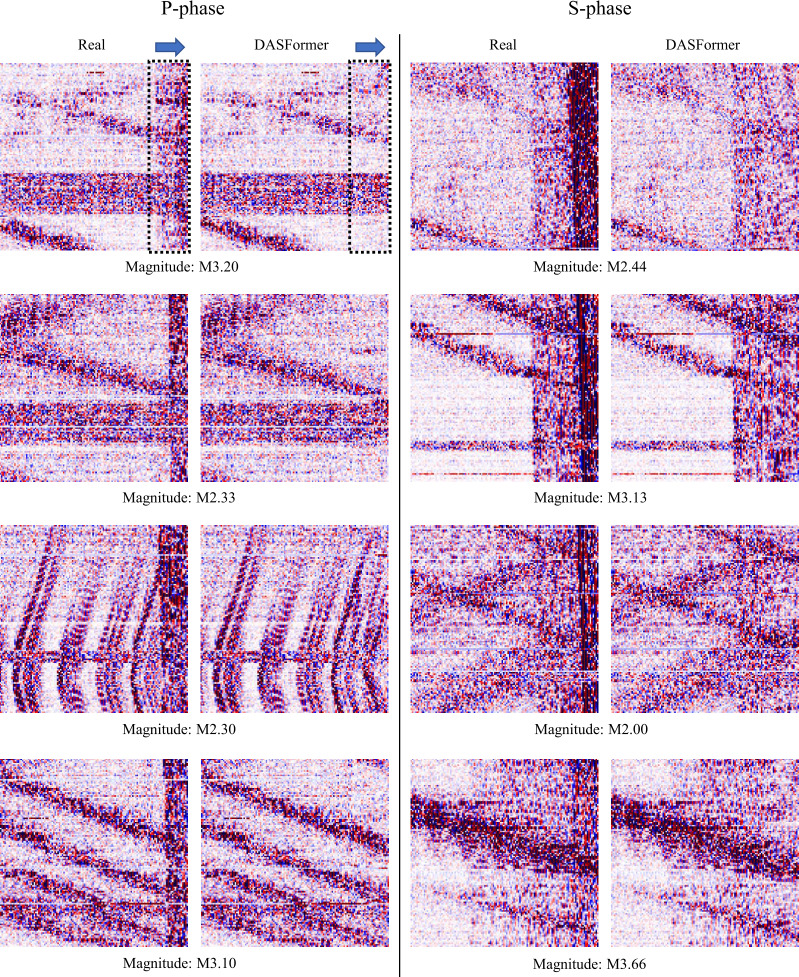
Visualization of forecasting results. The dashed frame and the blue arrow in the first case denote the forecasting window and direction, respectively, which are omitted in the rest of the cases for clarity. Our pre-trained DASFormer successfully detects all of these earthquake phases with high anomaly scores. Magnitude is from Southern California Earthquake Data Center (https://scedc.caltech.edu/)

In Fig. 12, we present eight more cases, illustrating the heightened precision of DASFormer in identifying patterns of traffic flow fluctuation over time. It is noteworthy how accurately DASFormer can detect both the ascending and descending trends in traffic flow.

Moreover, it is particularly proficient at identifying anomalies generated by defective sensors. These malfunctions, which can often misdirect analysis, are skillfully recognized and accounted for by DASFormer. This ability to discern erroneous data is crucial in maintaining the integrity of the overall analysis.

This dual capability of DASFormer – accurately predicting traffic flow patterns and adeptly identifying sensor malfunctions – results in robust P/S phase detections that significantly enhance the effectiveness of earthquake monitoring. This reinforces the reliability of DASFormer as a powerful tool in seismic studies, promising a more accurate understanding and prediction of earthquake patterns.

By integrating these capabilities, DASFormer is a highly dependable tool, providing comprehensive and accurate insights into traffic flow and sensor functionality. This, in turn, bolsters the robustness of P/S phase detections, thereby improving the accuracy and reliability of earthquake monitoring.

### Failure cases

**Figure 13:**
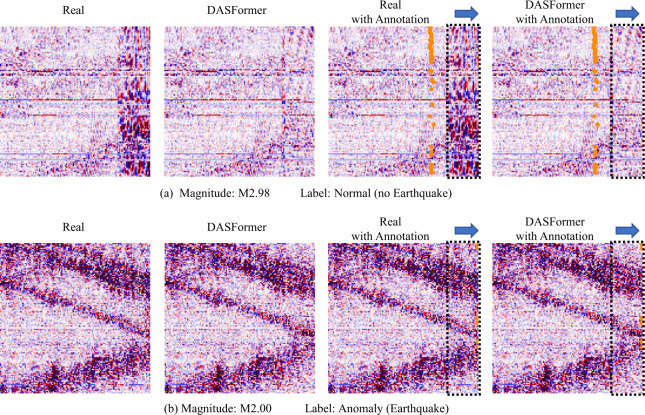
Visualization of failure cases. The dashed frame and the blue arrow in the first case denote the forecasting window and direction, respectively. The orange points indicate the data points annotated as P phase. (a) Inaccurate annotation. The sample should be labeled as an anomaly with earthquake. (b) Ambiguous annotation. The time period of earthquake signals in this sample is negligible to be labeled as an anomaly with earthquake

Remember that our labeling strategy designates a sample as an anomaly if a minimum of one data point in the forecast window is marked as a P/S phase. We highlight two instances of failure in Fig. 13. Upon examination, we can discern that these failures occur due to:

1. Inaccuracy in annotations. There can be discrepancies in the labeling process, leading to imprecise information that compromises the performance of the model.
2. Ambiguity in annotations. In some cases, the earthquake signals in the forecasting window are not substantial enough, yet these samples are marked as anomalies. The uncertainty in these instances makes it difficult for the model to identify anomalies correctly.

As a result, some instances that DASFormer accurately identifies are erroneously classified as false detections during the evaluation phase. This implies that DASFormer’s real-world performance may indeed be superior to what the evaluation metrics, such as ROC-AUC scores and F1 scores, suggest, as these metrics do not account for the unavoidable inaccuracies and ambiguities in the labeled samples.

In conclusion, our labeling strategy, while effective in most cases, has the potential to lead to misclassification. In spite of these challenges, DASFormer shows commendable resilience by still managing to detect many anomalies correctly.

### Time series view

**Figure 14:**
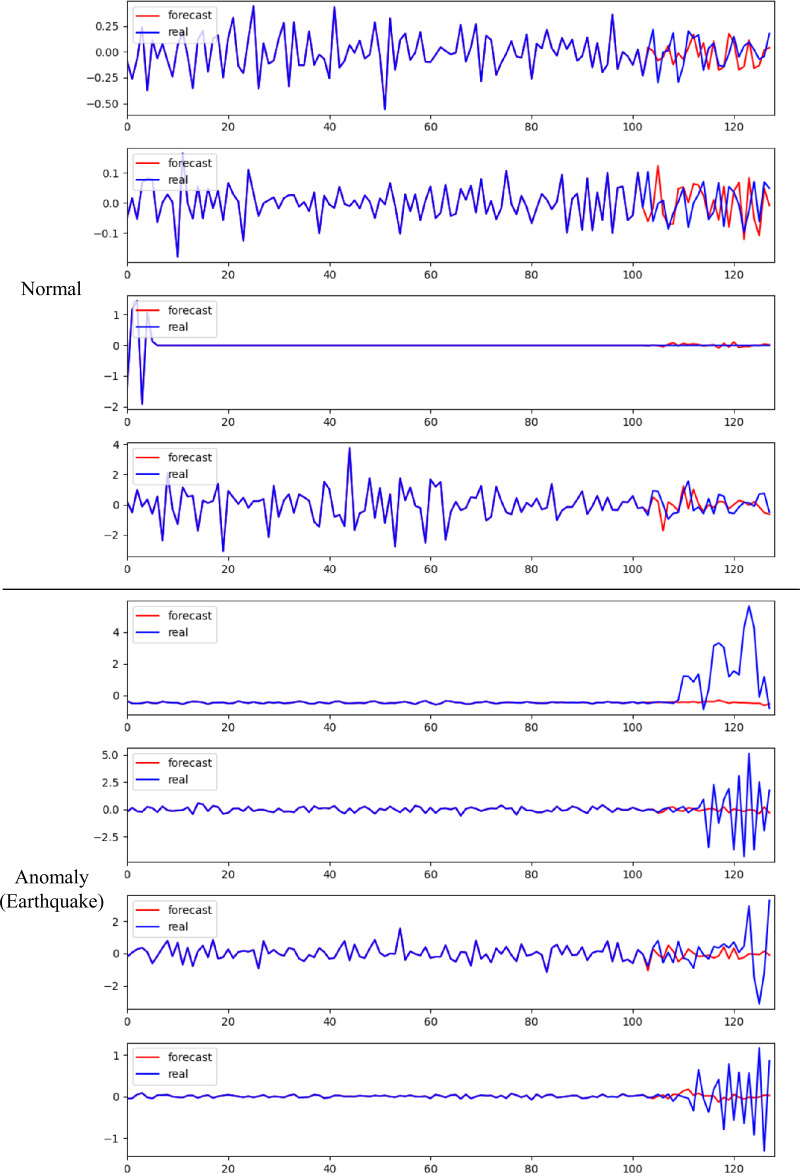
Illustration of results in a time series view. We randomly select 4 channels from normal cases and anomaly cases, respectively. The look-back and forecasting sizes are set to 103 and 25, respectively

We illustrate the results in a time series view in Fig. 14. As we can see, it is evident that DASFormer possesses the capability to accurately track the chronological trends and patterns observed in normal cases. Also, during instances of anomalies, the disparity between the predicted and actual values widens significantly, acting as a clear indicator of an impending earthquake.

The time series visualization provides a comprehensive picture of how DASFormer manages to interpret and represent the course of events over time. During regular, non-anomalous periods, the model successfully aligns its forecasts with the actual data, demonstrating its effectiveness in pattern recognition and trend analysis.

When it comes to anomalies, the model’s performance takes an interesting turn. As an earthquake approaches, the discrepancies between the forecasted data and the actual values become more pronounced. This suggests that DASFormer’s predictive accuracy decreases during these periods, but this very deviation serves as a critical signal of an upcoming seismic event. Thus, DASFormer not only excels in predicting normal conditions but also plays a vital role in earthquake detection, making it a crucial tool in the field of seismology.

## Conclusion

In this paper, we introduce DASFormer, a self-supervised framework for earthquake monitoring, which uses a coarse-to-fine structure with two U-Net architectures, allowing it to capture spatial and temporal correlations in DAS data. Our proposed features enhance the adaptation of visual models to multi-variate time series data. Our comparative analysis shows the superior performance of our method in unsupervised earthquake detection and tasks such as P/S phase picking and submarine pattern extraction. DASFormer paves the way for applying self-supervised learning to large-scale, unlabeled seismic datasets, representing a significant advance in earthquake monitoring.

## Data Availability

Ridgecrest DAS data is available on SCEDC AWS S3 bucket: s3://scedc-pds/Ridgecrest_DAS. The OOI North data is available at http://piweb.ooirsn.uw.edu/das.

## Footnotes

1 Ridgecrest DAS data is available on SCEDC AWS S3 bucket s3://scedc-pds/Ridgecrest_DAS.

2 The data is publicly available at http://piweb.ooirsn.uw.edu/das/.

## Abbreviations

AE: absolute error
ARIMA: autoregressive integrated moving average
BERT: bidirectional encoder representations from transformers
CNN: convolutional neural network
DAS: distributed acoustic sensing
EMD: earth mover’s distance
GAN: generative adversarial network
LSTM: long short-term memory
MLP: multi-layer perceptron
P/S phases: primary (p) and secondary (s) phases of seismic waves
RNN: recurrent neural network
ROC-AUC: receiver operating characteristic – area under curve
SMM: style manipulation module
STA/LTA: short-term averaging/long-term averaging for seismic signal detection
U-Net: convolutional network architecture for segmentation
VAE: variational autoencoder
ViT: vision transformer
